# Medical and Physical Intelligence

**Published:** 1806-07

**Authors:** 


### MEDICAL AND PHYSICAL INTELLIGENCE.

At a General Meeting of the Suffolk Benevolent Medical Society, held at Stow-market, on April 28, 1806, the following Resolutions were on the conclusion of the customary business of the Society, proposed by Dr. Drake, the President of the year; and after due consideration, unanimously adopted:

1st. That it appears to this society, that the proposed application to parliament, for a reform in the medical laws and institution of this country, is founded upon the most unquestionable necessity.

2dly. That the same irregularities and grievances in the practice of medicine, which have been pointed out with so much precision in the county of Lincoln, exist, though probably not to an equal extent, in the county of Suffolk.

3dly. That this society most decidedly approves of the plan, which shall, in future, subject medical men of every description to a regular education, and to subsequent examinations; convinced that the respectability of the profession, and therefore its utility, will be greatly promoted by a law of this kind emanating from the legislature.

4thly. It is of opinion, that, in future, a tax on decrees and certificates, somewhat similar to that which has been lately introduced into the legal profession, will, by excluding persons of a mean or low condition, have a direct tendency to increase the usefulness and dignity of a science, whose every department is so essentlally connected with the dearest interests of humanity.

5thly. It is of opinion, that Dr. Harrison has, by bringing forward the subject, deserved highly, not only of the medical profession, but of mankind at large; and this society unanimously resolves solves to give him every assistance in its power towards obtaining an object so truly desirable and important.

Signed by order of the Meeting,
RICHARD FREEMAN, Secretary.

*Hadleigh, Suffolk, May* 17, 1806.

---

M. HUMBOLDT lately read at a public meeting of the Academy of Sciences at Berlin, an interesting paper on the Physiognomy of Plants, which has been printed. The peculiar physiognomy he observed in the vegetable productions of the tropical countries, when compared with those of Europe, led Mr. H. to this classification.

Professor PFAFF, of Kiel, having been called to open the body of a person poisoned with arsenic, found that though it had lain fourteen days in the grave, putrefaction had not made any perceptible progress. This induced him to undertake an investigation thereof in a chemical point of view. From a comparison of the phenomena he here found, with those recorded in the works of other writers, he drew the conclusion, that arsenic, even when it remains a considerable time in the stomach, is not perceptibly dissolved, but, if it has been administered in substance, is again found there in that state, with hardly any loss, provided it has not been thrown up whilst the patient was alive. On the whole, it appears that arsenic is in a high degree insoluble; that of a large quantity left several weeks in contact with water, which was frequently poured over it, no evident solution took place. Of all the reagents or tests of its presence, he found water impregnated with sulphurated hydrogen gas the most active, insomuch that the presence of arsenic diffused in 60,000 times its weight of water was still betrayed by the well known yellow colour. The Professor considers a peculiar corrugation of the heart, especially on its anterior surface, is the surest sign of death having been caused by this poison.

Dr. KIDD has given an Analysis of a new mineral found in one of the Gwennah mines in Cornwall, and forming an incrustation round projecting particles of spongy pyrites intermixed with quartz. Its colour varied from a light ash to a dark brown; fracture like that of flint, presenting sections of concentric layers; texture close and polished like that of a nut, and of a silky lustre.— It is soluble in the nitric and muriatic acids with effervescence, violently decomposing the former, and giving out sulphurated hydrogen gas in abundance with the latter; and in both instances depositing a considerable proportion of sulphur. From an accurate series of experiments and analysis, this mineral appears to consist of about 33 parts of sulphur, and 66 of oxyd of zinc, with a very minute proportion of iron.

GAY LUSSAC has made further experiments relative to the discovery of Marechini, of fluoric acid in the enamel of the human teeth and in ivory. He found that the whole mass of ivory, and not merely the enamel, contains fluoric acid. He obtained the same same result from experiments with fossile ivory and the tusks of the wild boar. It is a remarkable fact, that as in the teeth the fluoric acid occurs in combination with the phosphoric acid, the phosphat of lime brought from Estremadura contains likewise fluat of lime, and that the earth brought from Marmoresch consists of much fluat of lime.

SIR GEORGE STAUNTON, having translated into the Chinese language a Treatise on the Vaccine Inoculation, (the first English work that ever was published in China), a general inoculation for the Cow-pox has taken place in the populous city of Canton. So far have this jealous people got the better of their prejudices in this instance, that a very large subscription was raised for establishing an institution in the city of Canton, by means of which the inoculation is to be spread into the neighbouring country, and the matter disseminated into every province of the empire.

A CHEMICAL SOCIETY is about to be established in London. The admission of subscribers is for the present limited to sixty, and the anuual subscription is fixed at three guineas. An unlimited number of gentlemen, residing in the country, may be admitted as subscribers, on paying one guinea annually, which shall entitle them to visit the Society as members, whenever they reside in the capital, provided their stay does not exceed three months. The admission of members is for the present confined to a Committee, who request, that such gentlemen as are desirous of becoming subscribers may favour them with their names, for which purpose a book is opened at their Laboratory, No. 11, Old Compton Street, Soho.

Mr. MURRAY, lecturer in chemistry, &c. at Edinburgh, has in the press a System of Chemistry, which will be published early in next winter.

---

Drs. BRADLEY and BATTY *have the satisfaction to announce to the Readers of the Medical and Physical Journal, that the Foreign Correspondence of this Work will in future be superintended by* Dr. PFAFF, *Professor of Medicine and Chemistry in the University of Kiel, a gentleman whose scientific character is highly respected in every part of Europe, and whose assistance cannot but add to the value and reputation of this Journal.*

*They cannot forbear to avail themselves of this occasion, to thank their numerous Correspondents in every part of the world, for the number, value, and originality of their favours; and as the circulation of the Medical Journal is as great at this time as at any period of its existence, those friends have the satisfaction to know, that their Communications enjoy the most extensive circulation at home and abroad, of which the press is susceptible.*

*For the information of several Correspondents, they think it proper to repeat, that the Indices to the first twenty Volumes are in course of preparation, and will appear soon after the completion of that Series.*

